# Retraction: Vault RNAs partially induces drug resistance of human tumor cells MCF-7 by binding to the RNA/DNA-binding protein PSF and inducing oncogene GAGE6

**DOI:** 10.1371/journal.pone.0300808

**Published:** 2024-03-26

**Authors:** The PLOS ONE Editors

Following the publication of this article [1], concerns were raised regarding results presented in Figs 1 and 3. Specifically,

- In Fig 1A, when adjusting the color levels to increase contrast, smears are visible in the background of the vtRNA1-2, vtRNA1-3, and vtRNA2-1 results.
- In Fig 1D there appears to be a vertical discontinuity between the PSF and DBD lanes, suggestive of a splice line.
- The following results presented in Fig 3B of [1] appear similar to results previously published in [2] under a CC-BY-4.0 DEED license by different authors, and results subsequently published in [3] by different authors:
  ○ The Fig 3B MC7 EdU panel (left) in [1], the MCF-7 PSF^KD^/vtRNA1-1 EdU panel (left) in [1], and the Fig 5A mimics PDGF-BB+ EdU panel in [3] when rotated 180°.
  ○ Fig 3B MCF-7-vtRNA 1–1 panels (left) in [1], Fig 4A MG63 panels in [2], and Fig 5A miR-NC PDGF-BB + panels in [3] when rotated 180°.
  ○ Fig 3B MCF-7 PSF^KD^/vtRNA1-1 panels (left) in [1] and Fig 5A mimics PDGF-BB+ panels in [3] when rotated 180°.
  ○ Fig 3B MCF-7-PSF^KD^ (left) panels in [1] and Fig 5A Vehicle PDGF-BB–panels in [3] when rotated 180°.
  ○ Fig 3B MCF-7 panels (right) in [1] and Fig 5A Vehicle PDGF-BB + panels in [3] when rotated 180°.
  ○ Fig 3B MCF-7-vtRNA1-1 panels (right) in [1] and Fig 5A inhibitors PDGF-BB + panels in [3] when rotated 180°.
  ○ Fig 3B MCF-7 vtRNA1-1/RBD panels (right) in [1] and Fig 4A MG63/vector panels in [2].
- In Fig 3B of [1], the MCF-7 EdU panel (left) appears similar to the MCF-7 PSF^KD^/vtRNA1-1 EdU panel (left).
- The Fig 3C left and right MCF-7-vtRNA1-1 panels appear similar.
- Contrary to the article’s Data Availability statement indicating that all relevant data are within the paper, the individual-level data underlying the published results have not been provided with the article.

The authors did not respond to editorial communications regarding the above concerns.

In light of the unresolved concerns that question the integrity and reliability of the reported data, the *PLOS ONE* Editors retract this article.

All authors either did not respond directly or could not be reached.
